# QNB: differential RNA methylation analysis for count-based small-sample sequencing data with a quad-negative binomial model

**DOI:** 10.1186/s12859-017-1808-4

**Published:** 2017-08-31

**Authors:** Lian Liu, Shao-Wu Zhang, Yufei Huang, Jia Meng

**Affiliations:** 1 Key Laboratory of Information Fusion Technology of Ministry of Education, School of Automation, Northwestern Polytechnical University, Xi’an, 710072 China; 2 Department of Electrical and Computation Engineering, University of Texas at San Antonio, San Antonio, TX 78230 USA; 3 Department of Biological Sciences, HRINU, SUERI, Xi’an Jiaotong-Liverpool University, Suzhou, Jiangsu 215123 China; 4 Institute of Integrative Biology, University of Liverpool, L7 8TX, Liverpool, UK

**Keywords:** Differential methylation analysis, m^6^A, Negative binomial distribution, RNA methylation, Small-sample size

## Abstract

**Background:**

As a newly emerged research area, RNA epigenetics has drawn increasing attention recently for the participation of RNA methylation and other modifications in a number of crucial biological processes. Thanks to high throughput sequencing techniques, such as, MeRIP-Seq, transcriptome-wide RNA methylation profile is now available in the form of count-based data, with which it is often of interests to study the dynamics at epitranscriptomic layer. However, the sample size of RNA methylation experiment is usually very small due to its costs; and additionally, there usually exist a large number of genes whose methylation level cannot be accurately estimated due to their low expression level, making differential RNA methylation analysis a difficult task.

**Results:**

We present QNB, a statistical approach for differential RNA methylation analysis with count-based small-sample sequencing data. Compared with previous approaches such as DRME model based on a statistical test covering the IP samples only with 2 negative binomial distributions, QNB is based on 4 independent negative binomial distributions with their variances and means linked by local regressions, and in the way, the input control samples are also properly taken care of. In addition, different from DRME approach, which relies only the input control sample only for estimating the background, QNB uses a more robust estimator for gene expression by combining information from both input and IP samples, which could largely improve the testing performance for very lowly expressed genes.

**Conclusion:**

QNB showed improved performance on both simulated and real MeRIP-Seq datasets when compared with competing algorithms. And the QNB model is also applicable to other datasets related RNA modifications, including but not limited to RNA bisulfite sequencing, m^1^A-Seq, Par-CLIP, RIP-Seq, etc.

**Electronic supplementary material:**

The online version of this article (10.1186/s12859-017-1808-4) contains supplementary material, which is available to authorized users.

## Background

**Fig. 1:**
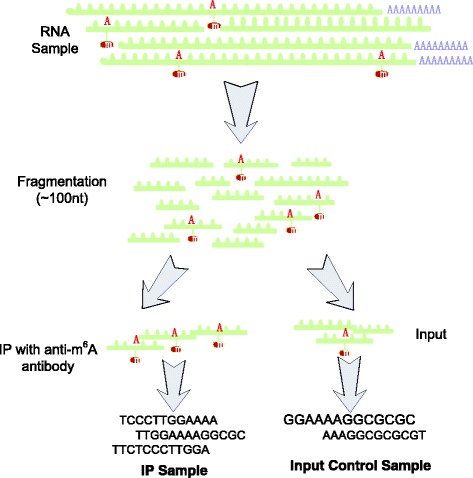
Illustration of MeRIP-Seq Protocol. In MeRIP-Seq, two types of samples (IP and input control samples) are generated. In the beginning of the protocol, RNA molecules are firstly sheared into fragments of around 100 nt. Through anti-m^6^A antibody, the IP sample provides unbiased measurement of the methylated RNA fragments; the input control sample reflects the basal RNA abundance

DNA chemical modifications and their functions have been well established through intensive research ranging from simple model organisms to human in the past decade [1–3]. While RNA modifications have yet drawn such attention until recent studies suggest RNA N6-methyladenosine (m^6^A) plays an important role in various biological processes, including circadian clock, RNA degradation, cocaine addiction, RNA-protein interaction, etc. [4, 5]. It is known that more than 100 different types of RNA modifications exist in all 3 kingdoms of life, and most of them are RNA methylation [6]. Till this day, the most widely applied approach for profiling transcriptome-wide RNA m^6^A methylation is methylated RNA immunoprecipitation sequencing (m^6^A-seq or MeRIP-seq), which combines methylated DNA immunoprecipitation (MeDIP), immunoprecipitation of RNA-binding proteins (RIP), and RNA sequencing (RNA-seq) to enable high-resolution detection of transcriptome-wide RNA methylation. MeRIP-Seq immunoprecipitates heavily fragmented, methylated RNA fragments with anti-m^6^A antibody and then sequences the purified RNA fragments for computational processing (See Fig. 1). Meanwhile, two types of samples, the IP and the input control, are obtained. The IP sample includes mostly the methylated fragments, while the input control sample includes all RNA fragments, which is generated to measure the basal RNA expression level of all genes as the background [7–9]. Different from whole exome sequencing (WXS), whole genome sequencing (WGS) and RNA-Seq, MeRIP-Seq needs anti-m^6^A antibody to capture the methylated mRNA fragments. In addition, due to the depleteon at both 5′ and 3′ ends as a result of RNA fragmentation and considerable variations in transcript abundance, it is necessary to have the input control sample. Till this day, MeRIP-Seq has been widely applied to various species, including, human, mouse, fly, pig, zebrafish, rice, yeast, HIV, etc., effectively unveiled the function of RNA m^6^A methylation in circadian clock, translation, miRNA processing, RNA-protein interaction, DNA damage response, etc. [10, 11]. However, due to the chemical instability of RNA molecule and the intricate experiment procedures, MeRIP-Seq experiment is still rather difficult to perform due to DNA contamination, RNA degradation or immunoprecipitation failure, etc.

By comparing the IP and input control samples, RNA methylation sites can be identified in a peak calling procedure [12, 13], based on which, differential RNA methylation analysis can unveil the dynamics in post-transcriptional RNA methylation under two different experimental conditions in a case-control study [14, 15].

Differential methylation analysis concerns the difference in methylation level between two conditions, which has shown to be of crucial biological significance [16]. Previously, there have been a number of computational approaches developed for differential methylation analysis of DNA [17–22]. Similar to DNA methylation, RNA methylation is also reversible and non-stoichiometric, and it is reasonable to speculate that the computational algorithms developed for DNA methylation are equally applicable to RNA methylation data. However, the unique features of RNA methylation and MeRIP-Seq technique call for novel computational approaches.

**Fig. 2:**
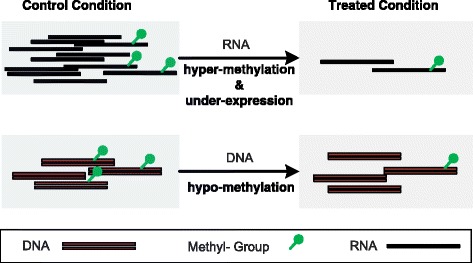
Differential methylation of DNA and RNA. Although the absolute amount of methylated RNA molecule decreases under the treated condition, the relative amount increased, indicating a hyper-methylation of the RNA molecule occurred together with expression down-regulation. In DNA methylation analysis, the absolute and relative amount of methylation always show consistent trend

The first important feature of MeRIP-Seq data is the highly heterogeous reads coverage due to different RNA expression level. When profiling the RNA methylome with MeRIP-Seq, the quantification of RNA methylation level usually starts from a paired integer measurements *t* and *c*, with *t* representing the number of reads proportional to the absolute amount of methylation and *c* proportional to the absolute amount of un-modified molecule. Specifically in MeRIP-Seq data, *t* refers to the reads count of a particular methylation site (or other feature) in the Immunoprecipitation (IP) sample, while *c* is calculated from the same site in the corresponding input control (input) sample. The methylation level*p* ∈ [0, 1] of this site can then be estimated by

1 $$ \widehat{p}=\frac{t}{t+c} $$

where $\widehat{p}$ denotes the percentage of methylation of this site on the corresponding RNA molecule. However, in practice, this estimation is not always accurate, e.g., although the same 100% of methylation is reported in two RNA methylation sites with measurements [*t*_1_, *c*_1_] = [100, 0] and [*t*_2_, *c*_2_] = [1, 0]. When sequencing noise is considered, the original reads count data of the two sites actually conveys substantially different information. While [*t*_1_, *c*_1_] = [100, 0]suggests a confident estimation of relatively high methylation level; [*t*_2_, *c*_2_] = [1, 0]essentially suggests that there is only very limited information received due to insufficient reads coverage, and the actual methylation level of this site is not accurately available. Conceivably, the estimation in Eq. (1) is relatively accurate only when *n* = *t* + *c* is large, which is often not true in RNA methylation sequencing data due to the existence of a large number of very lowly expressed genes. For this reason, a single estimated value for methylation level is usually not adequate for RNA methylation data processing, and it is necessary to keep the original integer measurements ( *t* and *c* ) for more precise quantification, which calls for count-based statistical models. Please note that, the aforementioned issue is different from the case of DNA methylation sequencing data, where a single value generated from Eq. (1) for the estimated methylation level is usually appropriate. This is because that the reads coverage of different CpG sites in DNA sequencing is usually highly homogeneous, so sufficient reads coverage can be reached simultaneously for most CpG sites of interests. Additionally, as shown in Fig. 2 **,** differential gene expression at RNA level may cause a discrepancy between the absolute amount of methylation and the relative amount, which calls for a precise estimation of the basal background and makes it different from the differential analysis of DNA methylation or DNA-protein interaction measured by ChIP-Seq.

The second prominent feature of MeRIP-Seq data is the limited number of samples (small sample size) available. Currently, due to the costs and technical difficulties of MeRIP-Seq experiment, there are usually no more than 3 biological replicates presented in a single study, which causes major difficulty in estimating the site-specific variability of RNA methylation level. When reliable estimation of variability in methylation level cannot be achieved, it is difficult to further assess whether the observed difference is due to within-group biological variability or not, making differential RNA methylation analysis between two experimental conditions fail. To solve this problem, we need novel approaches that work at even small-sample size scenario. Meanwhile, a number of small-sample inference approaches have been developed for sequencing data including, most notably, DESeq [23] and EdgeR [24], both of which rely on negative binomial distribution model with a linked variance and mean, which can shed light on this issue with a feasible solution for differential RNA methylation analysis problem at small sample size scenario.

To address the aforementioned limitations and challenges of MeRIP-Seq RNA methylation sequencing data, we propose here the QNB model, a small-sample size solution for differential RNA methylation analysis, which stands for quad-negative binomial model. With 4 cross-linked negative-binomial distributions for modeling the IP and Input control samples of MeRIP-Seq in two different experimental conditions, respectively, the proposed model is capable to robustly capture the within-group variability of RNA methylation level at small sample size scenario so as to perform more effective differential RNA methylation analysis. The model has been implemented in an R package that is freely available.

## Methods

**Fig. 3:**
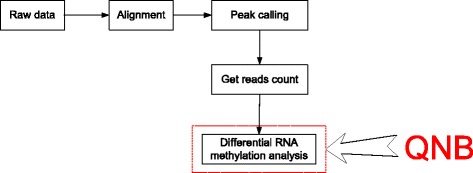
Differential RNA methylation data analysis. The complete differential RNA methylation analysis may require the following steps: reads alignment, peak calling (methylation site detection), reads counting and differential analysis

Differential RNA methylation data analysis includes the following steps: reads alignment, peak calling (methylation site detection), reads counting and differential analysis. The newly developed QNB package deals with the last step (See Fig. 3). Please note that, this is only one example. In practice, if differential methylation analysis is applied to gene or base resolution, only reads count is needed, and peak calling step will not be necessary.

### QNB model

Let *t*_*i* , *j*_ and *c*_*i* , *j*_ represent the reads counts of the *i*-th feature (gene or RNA methylation site) in the paired IP and input control sample of MeRIP-Seq data from *j*-th biological replicate, respectively. When the sequencing depths of different samples are the same, we may ignore its influence and have

2 $$ {t}_{i,j}\sim \mathrm{Binomial}\left({p}_{i,\rho (j)},{n}_{i,j}\right) $$

where *n*_*i* , *j*_ = *t*_*i* , *j*_ + *c*_*i* , *j*_ and *ρ*(*j*) represents the experimental condition (cell type, tissue or treatment) of the *j* -th biological replicate, and *p*_*i* , *ρ*(*j*)_ denotes the percentage of methylation for the *i* -th feature in *j* -th biological replicate. The goal of differential RNA methylation analysis for a specific feature is to test whether the percentage of methylation remain the same under two different experimental conditions $\mathcal{A}$ and ℬ, i.e., the null hypothesis ${p}_{i,\mathcal{A}}={p}_{i,\mathrm{\mathcal{B}}}$ .

Considering the over-dispersion effect of sequencing reads count data, *t*_*i* , *j*_and *c*_*i* , *j*_are assumed to follow the negative binomial distribution

3 $$ {t}_{i,j}\sim \mathrm{NB}\left({\mu}_{t,i,j},{\sigma}_{t,i,j}^2\right) $$

4 $$ {c}_{i,j}\sim \mathrm{NB}\left({\mu}_{c,i,j},{\sigma}_{c,i,j}^2\right) $$

where their means can be decomposed by

5 $$ {\mu}_{t,i,j}={q}_i{p}_{i,\rho (j)}{e}_{i,\rho (j)}{s}_{t,j} $$

6 $$ {\mu}_{c,i,j}={q}_i\left(1-{p}_{i,\rho (j)}\right){e}_{i,\rho (j)}{s}_{c,j} $$

Here, *q*_*i*_represents the expected abundance of feature *i* under all conditions in a standard sequencing library. *s*_*t* , *j*_and *s*_*c* , *j*_ represent the size factor of the IP and input control sample of the *j*-th biological replicate and directly reflect their sequencing depth. *p*_*i* , *ρ*(*j*)_ stands for risk of RNA methylation, or the true percentage of methylation for feature *i* under condition *ρ*(*j*) on the common scale, i.e., without rescaling by the size factors *s*_*c* , *j*_ and*s*_*t* , *j*_. Additionally, *e*_*i* , *ρ*(*j*) _is introduced to model differential expression at RNA level as a feature-specific size factor, which indicates the abundance of feature *i* under a specific experimental condition compared with the standard abundance *q*_*i*_.

In this model, the sequencing size factor *s*_*t* , *j*_ and *s*_*c* , *j*_ of the IP and input control sample can be conveniently estimated from the total number of the reads in a library or using the “geometric” approach developed for RNA-Seq data [23, 25]. The other parameters can be estimated as follows:

7 $$ {\widehat{q}}_i=\underset{\forall j}{\mathbb{E}}\left(\frac{t_{i,j}}{s_{t,j}}+\frac{c_{i,j}}{s_{c,j}}\right) $$

8 $$ {\widehat{p}}_{i,\rho (j)}=\sum_{j:\rho (j)=\rho}\left(\frac{t_{i,j}}{s_{t,j}}\right)/\sum_{j:\rho (j)=\rho}\left(\frac{t_{i,j}}{s_{t,j}}+\frac{c_{i,j}}{s_{c,j}}\right) $$

9 $$ {\widehat{e}}_{i,\rho }=\frac{1}{\left|\rho \right|{\widehat{q}}_i}\sum_{j:\rho (j)=\rho}\left(\frac{t_{i,j}}{s_{t,j}}+\frac{c_{i,j}}{s_{c,j}}\right) $$

where |*ρ*| denotes the number of biological replicates under a specific experimental condition *ρ* .

Please note that, compared with the DRME model [26], a more robust estimator for background expression level of the feature is implemented Eq. (7) by taking advantage of both the IP and input control samples. In DRME model, the basal level of gene expression is estimated from the input control sample only, as in theory without anti-body based enrichment, the input control sample of MeRIP-Seq data should contain both methylated and unmodified molecules, and thus corresponds to the true expression level. However, since the reads are usually enriched in the IP samples for a methylation sites to be called, there is usually less reads in the input control samples, and thus the estimator is not robust for very lowly expressed genes. For this reason, the basal level is estimated from the sum of input and IP samples in the QNB model. The robust estimator should largely improve the testing performance for very lowly expressed genes.

Inspired by the DESeq formulation [23], the variance in Eqs. **(** 3 **)** and **(** 4 **)** can be further decomposed into the shot noise and raw variance, i.e.,

10 $$ {\sigma}_{t,i,j}^2=\underset{\mathrm{shot}\  \mathrm{noise}}{\underbrace{\mu_{t,i,j}}}+\underset{\mathrm{raw}\ \mathrm{variance}}{\underbrace{{\left({e}_{i,j}{s}_{t,j}\right)}^2{\upsilon}_{t,i,\rho (j)}}} $$

11 $$ {\sigma}_{c,i,j}^2=\underset{\mathrm{shot}\  \mathrm{noise}}{\underbrace{\mu_{c,i,j}}}+\underset{\mathrm{raw}\ \mathrm{variance}}{\underbrace{{\left({e}_{i,j}{s}_{c,j}\right)}^2{\upsilon}_{c,i,\rho (j)}}} $$

where *μ*_*t* , *i* , *j*_ and *μ*_*c* , *i* , *j*_ are the variance of a Poisson distribution, which is often used to model technical replicates in NGS data. Additionally, due to biological variability, the over-dispersion of a Poisson model is represented by (*e*_*i* , *ρ*(*j*)_ *s*_*t* , *j*_)^2^ *υ*_*t* , *i* , *ρ*(*j*)_ and(*e*_*i* , *ρ*(*j*)_ *s*_*c* , *j*_)^2^ *υ*_*c* , *i* , *ρ*(*j*)_ , where *e*_*i* , *ρ*(*j*)_ and *s*_*t* , *j*_ (or *s*_*c* , *j*_ ) quantify the impact of condition-specific gene differential expression and sample-specific library size (or the sequencing depth), respectively. We consider the per-feature raw variance parameter *υ*_*i* , *ρ*_ is a smooth function of the expected methylation rate *p*_*i* , *ρ*_ and the feature abundance *q*_*i* , *ρ*_ under a specific condition *ρ* , i.e.,

12 $$ {\upsilon}_{t,i,\rho (j)}={\upsilon}_{t,\rho}\left({p}_{i,\rho (j)},{q}_{i,\rho (j)}\right) $$

13 $$ {\upsilon}_{c,i,\rho (j)}={\upsilon}_{c,\rho}\left({p}_{i,\rho (j)},{q}_{i,\rho (j)}\right) $$

For methylation reads count *t*_*i* , *j*_ in the IP sample, the variances on the common scale ${\widehat{w}}_{t,i,\rho }$ can be calculated with

14 $$ {\widehat{w}}_{t,i,\rho }=\frac{1}{\left(\left|\rho \right|-1\right)}\sum_{j:\rho (j)=\rho }{\left[\frac{t_{i,j}}{{\widehat{s}}_{t,j}{\widehat{e}}_{i,\rho (j)}}-{\overline{q}}_{t,i,\rho}\right]}^2 $$

where

15 $$ {\overline{q}}_{t,i,\rho }=\frac{1}{\left|\rho \right|}\sum_{j:\rho (j)=\rho}\frac{t_{i,j}}{{\widehat{s}}_{t,j}{\widehat{e}}_{i,\rho (j)}} $$

Let

16 $$ {z}_{t,i,\rho }=\frac{{\widehat{q}}_i{\widehat{p}}_{i,\rho (j)}}{\left|\rho \right|}\sum_{j:\rho (j)=\rho}\left(\frac{1}{{\widehat{s}}_{t,j}{\widehat{e}}_{i,\rho (j)}}\right) $$

Following the methodology of DESeq model [23], we show in the supplementary materials (Additional file 1) that$\left({\widehat{w}}_{t,i,\rho }-{z}_{t,i,\rho}\right)$ is an unbiased estimator for the raw variance parameter *υ*_*t* , *i* , *ρ*_, with

17 $$ {\widehat{\upsilon}}_{t,i,\rho (j)}\left({\widehat{p}}_{i,\rho },{\widehat{q}}_i\right)={w}_{t,i,\rho}\left({\widehat{p}}_{i,\rho },{\widehat{q}}_i\right)-{z}_{t,i,\rho } $$

as our estimate for the raw variance parameter *υ*_*t* , *i* , *ρ*(*j*)_.

We use a 2-dimensional local regression on the graph $\left({\widehat{p}}_{i,\rho },{\widehat{q}}_i,{\widehat{w}}_{t,i,\rho}\right)$to obtain a smooth function of${w}_{t,i,\rho}\left({\widehat{p}}_{i,\rho },{\widehat{q}}_i\right)$. Since ${\widehat{w}}_{t,i,\rho }$in Eq. **(** 14 **)** is the sum of squared random variable, the residuals of the model${w}_{t,i,\rho }-{w}_{t,i,\rho}\left({\widehat{p}}_{i,\rho },{\widehat{q}}_{i,\rho}\right)$ are skewed. Following reference [27] and the practice in DESeq [23], we also implemented a generalized linear model of the gamma family for the local regression with the implementation in R locfit package [28] for estimation of ${w}_{t,i,\rho}\left({\widehat{p}}_{i,\rho },{\widehat{q}}_i\right)$.

Similar to the estimation of *υ*_*t* , *i* , *ρ*(*j*)_ and *w*_*t* , *i* , *ρ*_ in the IP samples as described previously, the raw variance parameter *υ*_*c* , *i* , *ρ*(*j*)_ and the variance of reads on the common scale *w*_*c* , *i* , *ρ*_ for the input control samples can also be estimated.

### Testing & Metrics

For differential RNA methylation analysis, we consider the null hypothesis that condition $\mathcal{A}$ and condition ℬ are of the same methylation rate on the common scale, i.e.,${p}_{i,\mathcal{A}}={p}_{i,\mathrm{\mathcal{B}}}={p}_{i,\mathcal{O}}$, which can be estimated with

18 $$ {\widehat{p}}_{i,\mathcal{O}}=\sum_{j\in \mathcal{A}\cup \mathrm{\mathcal{B}}}\frac{t_{i,j}}{s_{t,j}}/\sum_{j\in \mathcal{A}\cup \mathrm{\mathcal{B}}}\left(\frac{t_{i,j}}{s_{t,j}}+\frac{c_{i,j}}{s_{c,j}}\right) $$

For each feature *i* and replicate *j *of its condition *ρ*(*j*), the reads counts *t*_*i* , *j*_and *c*_*i* , *j*_are considered independently distributed. For differential methylation analysis between condition $\mathcal{A}$ and ℬ, we construct 4 random variables following negative binomial distributions for the IP and input control samples under two experimental conditions, respectively, i.e.,

19 $$ {t}_{i,\mathcal{A}}=\sum_{j\in \mathcal{A}}\left({t}_{i,j}\right)\sim \mathrm{NB}\left({\widehat{\mu}}_{t,i,\mathcal{A}},{\widehat{\sigma}}_{t,i,\mathcal{A}}^2\right) $$

20 $$ {t}_{i,\mathrm{\mathcal{B}}}=\sum_{j\in \mathrm{\mathcal{B}}}\left({t}_{i,j}\right)\sim \mathrm{NB}\left({\widehat{\mu}}_{t,i,\mathrm{\mathcal{B}}},{\widehat{\sigma}}_{t,i,\mathrm{\mathcal{B}}}^2\right) $$

21 $$ {c}_{i,\mathcal{A}}=\sum_{j\in \mathcal{A}}\left({c}_{i,j}\right)\sim \mathrm{NB}\left({\widehat{\mu}}_{c,i,\mathcal{A}},{\widehat{\sigma}}_{c,i,\mathcal{A}}^2\right) $$

22 $$ {c}_{i,\mathrm{\mathcal{B}}}=\sum_{j\in \mathrm{\mathcal{B}}}\left({c}_{i,j}\right)\sim \mathrm{NB}\left({\widehat{\mu}}_{c,i,\mathrm{\mathcal{B}}},{\widehat{\sigma}}_{c,i,\mathrm{\mathcal{B}}}^2\right) $$

It is not difficult to calculate the distribution parameters in Eqs. (19), (20), (21) and (20). Taking ${t}_{i,\mathcal{A}}$for example, we have

23 $$ {\widehat{\mu}}_{t,i,\mathcal{A}}={\widehat{p}}_{i,\mathcal{O}}{\widehat{q}}_i{\widehat{e}}_{i,\mathcal{A}}\sum_{j\in \mathcal{A}}{\widehat{s}}_{t,j} $$

24 $$ {\widehat{\sigma}}_{t,i,\mathcal{A}}^2={\widehat{p}}_{i,\mathcal{O}}{\widehat{q}}_i{\widehat{e}}_{i,\mathcal{A}}\sum_{j\in \mathcal{A}}{s}_{t,j}+{\upsilon}_{\mathcal{A}}\left({\widehat{p}}_{i,\mathcal{O}},{\widehat{q}}_i\right){\widehat{e}}_{i,\mathcal{A}}^2\sum_{j\in \mathcal{A}}{\widehat{s}}_{t,j}^2 $$

Given the total number of methylation read count $\left({t}_i={t}_{i,\mathcal{A}}+{t}_{i,\mathrm{\mathcal{B}}}\right)$ and the total number of reads under each condition $\left({n}_{i,\mathcal{A}}={t}_{i,\mathcal{A}}+{c}_{i,\mathcal{A}}\right)$ and (*n*_*i* , ℬ_ = *t*_*i* , ℬ_ + *c*_*i* , ℬ_) do not change, the joint conditional probability of the observation $\left({t}_{i,\mathcal{A}}=t\right)$ can be calculated with

25 $$ {\displaystyle \begin{array}{c}P\left({t}_{i,\mathcal{A}}=t|{t}_i,{n}_{i,\mathcal{A}},{n}_{i,\mathrm{\mathcal{B}}}\right)=P\left({t}_{i,\mathcal{A}}=t\right)P\left({t}_{i,\mathrm{\mathcal{B}}}={t}_i-t\right)\\ {}P\left({c}_{i,\mathcal{A}}={n}_{i,\mathcal{A}}-t\right)P\left({c}_{i,\mathrm{\mathcal{B}}}={n}_{i,\mathrm{\mathcal{B}}}-{t}_i+t\right)\end{array}} $$

whose components are previously defined in Eqs. (19), (20), (21) and (22).

Please note that, the over-dispersion of reads counts in input control samples are also modeled and covered in the QNB test, making it substantially different from the DESeq, DRME or ChIPComp. The QNB test essentially covers all the 4 samples with 4 cross-linked binomial distributions; while in DRME model, the input control samples are used only for gene expression estimation, so the statistical test covers the IP samples only with 2 negative binomial distributions. The inclusion of input control samples in the test, rather than simply using it as a background, makes a major contribution to the performance improvement, and also makes QNB substantially different from all other count-based (negative-binomial distribution-based) approaches such as DRME, edgeR, DESeq and ChIPComp.

The statistical significance of an observation can then be calculated using a two-sided test

26 $$ p\hbox{-} \mathrm{value}=\frac{\sum_{t:P(t)\le P\left({t}_{i,\mathcal{A}}\right)}P(t)}{\sum_{\forall t}P(t)} $$

Besides the *p*-value that quantifies the statistical significance, the risk ratio (RR) of RNA methylation level, which quantifies the degree of differential methylation, can also be calculated based on Eq. (8), with

27 $$ {\mathrm{RR}}_i={\widehat{p}}_{i,\mathcal{A}}/{\widehat{p}}_{i,\mathrm{\mathcal{B}}} $$

where conditionℬ is considered as the control group in a case-control study and $\mathcal{A}$ as the treated group. Please note that, the percentage of methylation under an experimental condition ${p}_{i,\mathcal{A}}$ denotes a normalized degree of methylation observed on the data rather than the true percentage of methylation in biological sense. However, it still provides a good evaluation of the relative methylation level. Similar to the methylation risk ratio (RR), the odds ratio (OR) of RNA methylation, which also quantifies the degree of differential RNA methylation, can be calculated after compensating the sample sequencing depth

28 $$ {\mathrm{OR}}_i=\left\{\frac{\sum_{j\in \mathcal{A}}\left({t}_{i,j}/{s}_{t,j}\right)}{\sum_{j\in \mathcal{A}}\left({c}_{i,j}/{s}_{c,j}\right)}\right\}/\left\{\frac{\sum_{j\in \mathrm{\mathcal{B}}}\left({t}_{i,j}/{s}_{t,j}\right)}{\sum_{j\in \mathrm{\mathcal{B}}}\left({c}_{i,j}/{s}_{c,j}\right)}\right\} $$

## QNB package

The proposed method has been implemented in the QNB R package and is freely available through the Comprehensive R Archive Network (CRAN): https://cran.rstudio.com/web/packages/QNB/. For sample size factor estimation, QNB uses the “geometric” approach [23, 25] by default, but it is also possible for the user to provide the size factors calculated from other methods. It is also worth mentioning that, compared with the DRME model, QNB package allows 4 different modes for estimating the raw variance parameter in Eq. (17) for different scenarios, including, “per-condition”, “pooled”, “blind” and “auto”.

- The mode “per-condition” calculates an empirical dispersion value by considering the data from samples for this condition for each condition with replicates.
- The mode “pooled” estimates a single pooled dispersion value using the samples from all conditions with replicates.
- The mode “blind” ignores the sample labels and estimates a dispersion value as if all samples were replicates of a single condition, so this mode supports variance estimation even if there are no real biological replicates from the same condition available.
- The mode “auto” selects mode according to the number of samples automatically. Under this option, “per-condition” mode is adopted when biological replicates are available for a more sensitive estimation of the raw variance parameter; while the “blind” mode is used when no biological replicates are available.

QNB package implements the “auto” mode by default.

## Results

To evaluate the performance of the proposed method, it is tested on simulated and real datasets, and compared with other approaches including exomePeak [12], MeTDiff [15], DRME [26] and Bltest [29]. We have also included in the comparison the DSS method [30], which is a most recent method developed for DNA differential methylation analysis, and the ChIPComp method [31], which was developed for differential binding analysis from ChIP-Seq data.

### Test on simulated dataset

The simulated data mimics the reads count information of 20,000 methylation sites in 3 IP and input control samples from two experimental conditions. Specifically, to simulate the impact of differential expression, we let log(*q*_*i*_) follow a uniform distribution and the percentage of methylation *p*_*i* , *ρ*(*j*)_ follow a uniform distribution between 0 and 1. The two size factors *e*_*i* , *ρ*(*j*)_ and *s*_*t* , *j*_ are set to follow normal distributions after log transformation, in which the variance can be adjusted to mimic the impact of condition-specific differential expression and different sequencing depth. In addition, *p*_*i* , *ρ*(*j*)_ are set to be equal between two conditions for 50% of the RNA methylation sites, which are corresponding to the non-differential sites. The others are set different as the true differential RNA methylation sites. Additionally, we set *υ*_*t* , *i* , *ρ*(*j*)_ = *d*/{*e*_*i* , *ρ*(*j*)_ *s*_*t* , *j*_} and *υ*_*c* , *i* , *ρ*(*j*)_ = *d*/{*e*_*i* , *ρ*(*j*)_ *s*_*c* , *j*_}to mimic the impact of over-dispersion among biological replicates. Here, *d* is a constant value to quantify the degree of over-dispersion, with a greater value indicating increased difference among biological replicates from the same condition. To evaluate the performance of the methods tested, 100 random datasets are generated and tested against these methods, and their area under receiver operating characteristic curves (AUCs) are calculated to evaluate their performance, respectively.

**Fig. 4:**
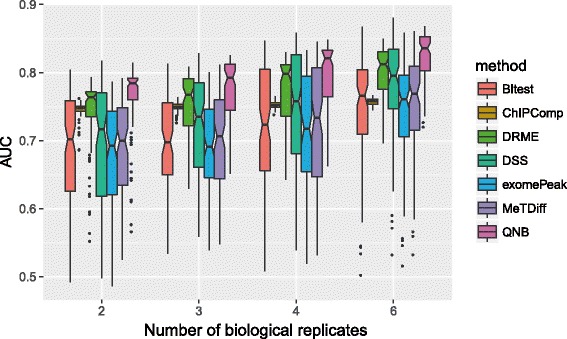
Impact from number of biological replicates on differential RNA methylation analysis. The performance of all 7 methods tested increases as the number of biological replicates increases, suggesting biological replicates are still essential for the proposed small-sample inference approach. QNB method outperforms competing approaches on datasets with 2, 3, 4 and 6 biological replicates, succeeded by DRME, DSS and ChIPComp

In the first experiment, we tested the impact from the number of biological replicates on the performance of differential RNA methylation analysis. As shown from Fig. 4, when the number of biological replicates increases, the performance of all 7 approaches increases. This is reasonable as additional information is provided when the number of biological replicates increases. The proposed QNB method consistently outperforms the competing methods on datasets with 2, 3, 4 or 6 biological replicates; however, sufficient number of biological replicates is still essential for more reliable results.

**Fig. 5:**
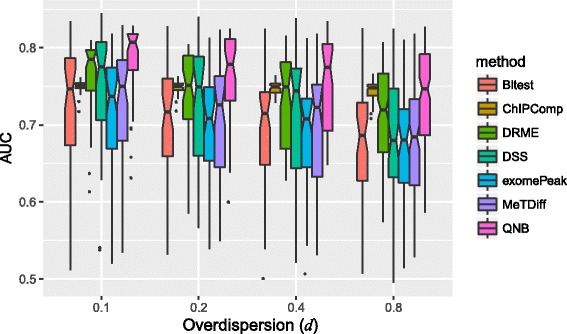
Impact of over-dispersion on differential RNA methylation analysis. The performance of differential RNA methylation decreases as the over-dispersion increases, and QNB method consistently outperforms the competing methods, succeeded by DRME, DSS and ChIPComp

We then tested the impact of over-dispersion on the differential RNA methylation performance. As shown in Eqs. (10) and (11), over-dispersion is directly tied up with the variance of reads count, so it is not surprising to see from Fig. 5 that, the performance of all 7 approaches decreases as over-dispersion increases. Specifically, QNB method still consistently outperforms the competing methods on different dispersion settings tested.

**Fig. 6:**
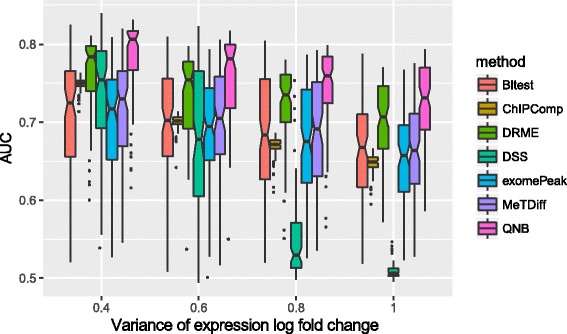
Impact of RNA differential expression on differential RNA methylation analysis. In this experiment, we adjusted the variance of *e*_*i* , *ρ*(*j*)_ for the impact of differential expression setting. It can be seen that, the performance of differential RNA methylation analysis decreases as the degree of differential expression increases, and QNB achieved better performance than competing approaches under all 4 setting tested

In the 3rd experiment, we tested the impact of differential expression, which contributed to a major difference between RNA and DNA methylation analysis. As shown in Fig. 6, changes in expression level between different conditions hinder the performance of differential RNA methylation analysis, which is reasonable because it leads to unbalanced reads count in two experimental conditions, i.e., a lot of reads under one condition but very limited number of reads under the other condition. QNB can handle differential expression relatively well and perform better than the competing methods.

### Test on human U2OS dataset

QNB approach was then tested on real RNA methylation sequencing dataset that profiles m^6^A methylome in untreated U2OS cells and after treated with SAH hydrolysis inhibitor 3-deazaadenosine (DAA) [32]. The original raw data in SRA format was obtained directly from GEO (GSE48037), which consists of 3 IP and 3 Input MeRIP-Seq replicates under control condition and after DAA treatment, respectively (a total of 12 libraries). The short sequencing reads are firstly aligned to human genome assembly hg19 with Tophat2 [33]. In the reads alignment step, other splice-aware aligners such as Tophat2 [33], HISAT [34], STAR [35], RSEM [36], Kallisto [37] and Salmon [38] are also applicable. Then, a total 29,427 RNA N6-methyl-adenosine (m^6^A) sites are called by using exomePeak R/Bioconductor package with UCSC gene annotation database. In the peak calling step, to obtain a consensus RNA methylation site set between two experimental conditions (control and DAA treatment), the IP and Input control samples are merged, respectively. Then we used Bioconductor packages GenomicFeatures and Rsamtools [39] on R platform to obtain the reads count of every RNA methylation sites from the 3 IP and input control samples under two conditions, respectively. The reads count information can then be used for comparing QNB method with the other competing approaches.

**Fig. 7:**
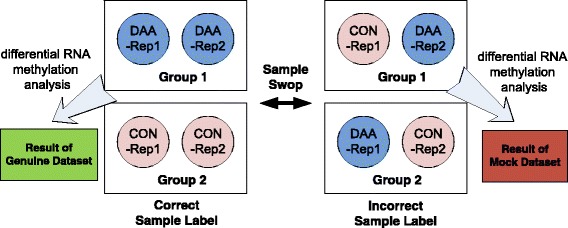
Creation of the mock dataset with sample swop. A “mock” dataset can be created from the original dataset by swop half of the samples between the two experimental conditions. The differential RNA methylation result generated from the original data with correct sample label reflects biological meaningful difference; while the result generated from the “mock” dataset has no biological meaning. In theory, a good algorithm should pick up as many as differential methylation sites from the “genuine” dataset but as less as differential methylation sites from the “mock” dataset. The example above shows how a pair of “genuine” and “mock” datasets is created from two biological replicates - sample 1 and sample 2. Since the tested MeRIP-Seq dataset has 3 biological replicates under each condition, it is possible to create 3 pairs of “genuine” and “mock” datasets from 3 pairs of replicates, i.e., sample 1 and 2, sample 2 and 3, sample 3 and 1. It is then possible to compare the performance of different algorithms

A major limitation for testing differential RNA methylation analysis with real dataset is the lack of experimentally validated true differential methylation site. Without ground truth, it is difficult to effectively compare the performance of different approaches. For this reason, we designed a sample-swop test by taking advantage of a set of true negative data generated by sample swop. In the designed sample-swop test, differential RNA methylation analysis is firstly conducted on the original data with correct sample class label information and generated a set of“genuine”result; then differential analysis is applied to a “mock” dataset with half of the samples swopped between the two conditions tested to generate a set of “mock” result. Compared with the “genuine” result that is expected to carry biological meaning, the “mock” result is generated with incorrect sample labels and thus represents a background associated with no biological meanings (see Fig. 7). For the aforementioned reasons, an effective differential RNA methylation method should report as many differential methylation sites as possible in the “genuine” result, and at the same time report as less differential methylation sites as possible in the “mock” result given a specific confidence level. In another word, when two approaches report the same number of DRMSs on the “mock” dataset, the one that reports more DRMSs on the “genuine” dataset achieved a better performance.

**Fig. 8:**
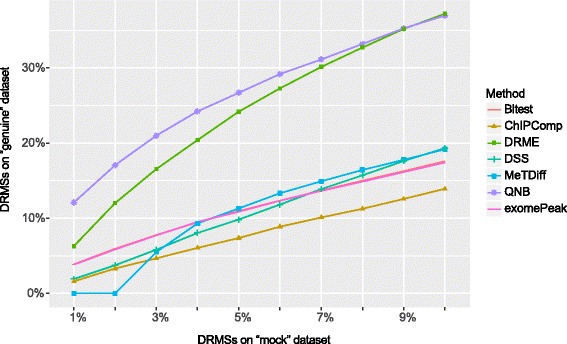
Comparison of differential algorithms on human DAA treatment experiment with sample-swop test. We generated 3 pairs of “genuine” and “mock” datasets with the 3 biological replicates from the control and DAA treatment MeRIP-Seq experiment. By fixing the percentage of DRMSs in the 3 “mock” datasets, we calculated the percentage of DRMSs in their corresponding “genuine” datasets at the same significance level. QNB outperforms the competing methods especially at high significance level. The exomePeak method and Bltest achieved almost the same performance

As is shown in Fig. 8, QNB outperforms the other competing algorithm on real MeRIP-Seq dataset in the sample-swop tests, especially at more stringent significance level. In the figure, x-axis represents the percentage of DRMSs called on “mock” dataset, and y-axis represents the percentage of DRMSs detected on the corresponding “genuine” datasets. For QNB approach, when 1% of sites are reported as DRMSs on “mock” datasets, around 12% of DRMSs are reported on the corresponding “genuine” datasets. With an assumption that there exists similar background noise in “mock” and “genuine” datasets, the DRMSs reported in the “genuine” dataset should have a false discovery rate of around 0.073. Please note that, in the sample swop test above, a negative dataset was created when positive data is not available. Similar strategies have been used previously [13, 15, 40].

**Fig. 9:**
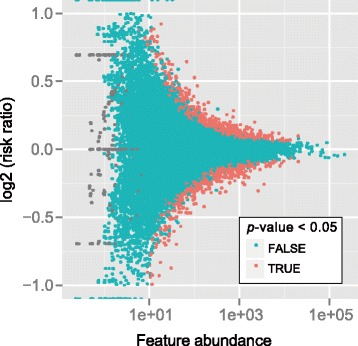
Differential RNA methylation analysis. QNB method identified 1355 DRMSs out of a total of 29,427 RNA methylation sites after DAA treatment to U2OS cells at significance level 0.05. Compared with the features with less number of reads, the observed methylation fold changes for abundant features have a smaller range, and the DRMSs identified are mostly with larger methylation risk ratio between the two conditions compared with the features of a similar abundance

We then applied the QNB method to the complete MeRIP-Seq dataset including all the replicates. In the end, 1355 out of 29,427 RNA methylation sites are identified as DRMSs at significance level 0.05 by QNB method. As shown in Fig. 9, the DRMSs identified by QNB method are mostly with large methylation risk ratio compared with the features of a similar abundance.

### Test on mouse midbrain dataset

We showed previously with a sample-swop test that, QNB method outperforms competing methods on a real RNA methylation sequencing dataset that profiles the epitranscriptomic impact of DAA treatment to human U2OS cells. It is necessary to examine whether this is still true on a different dataset. For this purpose, we repeated this test on a different MeRIP-Seq dataset, which studies the impact of FTO knock down in mouse midbrain [41].

**Fig. 10:**
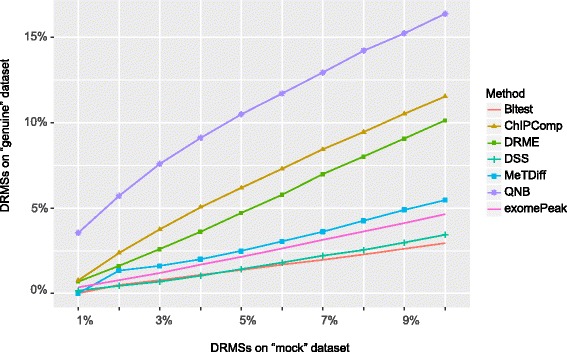
Comparison of differential algorithms on mouse FTO knock down experiment with sample-swop test. Result suggests that, QNB outperforms the competing methods especially at high significance level, succeeded by ChIPComp, DRME and MetDiff. However, different from the human U2OS dataset, exomePeak and Bltest methods do not behave similarly on this dataset

Similar settings are adopted as previously described in the human dataset. The sequencing reads are downloaded from NCBI GEO and then aligned to mouse mm10 genome assembly with Tophat2 aligner, then R/Bioconductor packages are used for identifying the RNA methylation sites and counting the number of reads associated with them. Similar to the DAA treatment experiment described previously, 3 pairs of “genuine” and “mock” datasets are generated with the 3 biological replicates from the control and FTO knock down MeRIP-Seq experiment. By fixing the percentage of differential RNA methylation sites (DRMSs) in the 3 “mock” datasets, we calculated the percentage of DRMSs in their corresponding “genuine” datasets at the same significance level. It can be seen from Fig. 10 that, QNB outperforms the competing approaches in the sample-swop test on this mouse MeRIP-Seq dataset, especially at more stringent significance level.

## Discussion

The newly proposed approach is in many ways related to DESeq sand DRME model, including the negative binomial assumption of reads count data, the decomposition of variance into the shot noise and the raw variance, the usage of local regression of gamma family for estimating the variance and the construction of the test; however, QNB also extended these two models by including the input control samples as additional components for a more comprehensive statistical evaluation. And compared with the DRME method [26], a more robust estimator of the background (RNA expression level) is used by merging information from both the IP and input control samples. Importantly, as shown on simulated system and the real MeRIP-Seq datasets from human and mouse, we showed in a sample-swop test that, QNB obviously outperforms the existing differential RNA methylation approaches, including exomePeak [12], MeTDiff [15], DRME [26] and Bltest [29]. It also outperforms DSS [30], a method developed for DNA methylation differential analysis, and ChIPComp [31], a method developed for ChIP-Seq analysis.

There exist a number of issues that may affect the performance of QNB method in differential RNA methylation analysis. Firstly, biological replicates are still essential for achieving reliable results. As shown in Fig. 4, increased number of replicates helps to improve the prediction performance of QNB and the other 6 methods tested. Secondly, due to the existence of very lowly expressed genes, adequate sequencing depth is still necessary for detecting the features of low abundance. Thirdly, QNB relies on accurate reads count data of the RNA methylation sites (or other features), so precise determination of RNA methylation sites on the transcripts and proper sequencing reads alignment and counting are indispensable. In MeRIP-Seq data, it can be difficult to differentiate isoform transcripts and thus difficult to perform isoform-specific differential RNA methylation analysis. Fourthly, data quality can still be a major limitation for RNA methylation sequencing experiments because of the technical difficulties and high costs. Without proper experiment design and implementation, the following computational analyses may end in vain. Fifthly, it is still an open question how to best estimate the library size factor of the samples for MeRIP-Seq data. Conceivably, the size factors of the IP and input control samples may not be directly comparable due to their instinct properties and their distinct distribution patterns, and the immunoprecipitation efficiency of different IP samples may not be the same. Sixthly, the proposed method assumes that the variability of methylation level is a smooth function of expression level and methylation level; however, as the number of biological replicates increases, a more straightforward approach might be directly modeled and estimate site-specific variability without this assumption. All the aforementioned issues call for further investigation and improvements.

## Conclusions

RNA methylation has emerged as an important layer for gene regulation, where biological functions are modulated by reversible post-transcriptional RNA modifications. We proposed here a QNB model together with an R package for differential RNA methylation analysis at small sample size scenario. The method is based on four negative binomial distributions with their means and variances cross-linked together, which model the IP and input control samples under 2 experimental conditions, respectively. Compared with other methods on the simulated and real MeRIP-Seq datasets, QNB is much more effective for differential RNA methylation analysis with the small-sample sequencing data. QNB model can also be applied to other data types related to RNA modifications, such as RNA bisulfite sequencing, m^1^A-Seq, Par-CLIP and RIP-Seq.

## Additional file

Additional file 1: Proof: $\left({\widehat{w}}_{t,i,\rho }-{z}_{t,i,\rho}\right)$ is an unbiased estimator for *υ*_*t* , *i* , *ρ*_. (PDF 383 kb)

## Abbreviations

DRMS: differential RNA methylation sites
IP: the immunoprecipitation
m^1^A: N1-methyladenosine
m^6^A: N6-methyladenosine
MeRIP-Seq: methylated RNA immunoprecipitation sequencing
NB: the negative binomial distribution
OR: the odds ratio
Par-CLIP: photoactivatable ribonucleoside-enhanced crosslinking and immunoprecipitation
QNB: quad-negative binomial model
RIP-Seq: RNA Immunoprecipitation sequencing
RR: the risk ratio
