# Supplementary material for: QNB: differential RNA methylation analysis for count-based small-sample sequencing data with a quad-negative binomial model
Source: BMC Bioinformatics. 2017 Aug 31;18:387. doi: 10.1186/s12859-017-1808-4 (PMC5667504; doi:10.1186/s12859-017-1808-4)
Supplement: Additional file 1: — Proof: $\left({\widehat{w}}_{t,i,\rho }-{z}_{t,i,\rho}\right)$w^t,i,ρ−zt,i,ρ is an unbiased estimator for υ t , i , ρ. (PDF 383 kb) [file 12859_2017_1808_MOESM1_ESM.pdf]

Proof: $\left(\hat{w}_{t,i,\rho} - z_{t,i,\rho}\right)$ is an unbiased estimator for $\upsilon_{t,i,\rho}$

The following derivation was inspired by the DESeq model [1]. Following the notation in the main text, let $q_{t,i,j} = \dfrac{t_{i,j}}{\hat{s}_{t,j}\hat{e}_{i,\rho(j)}}$, then we have,

$$\begin{aligned}(|\rho|-1)\hat{w}_{t,i,\rho} &= \sum_{j:\rho(j)=\rho}\left[q_{t,i,j} - \bar{q}_{t,i,\rho}\right]^2 \\ &= \sum_{j:\rho(j)=\rho}\left[\left(q_{t,i,j}\right)^2 - \left[\frac{2}{|\rho|}q_{t,i,j}^2 + \frac{2}{|\rho|}q_{t,i,j}\sum_{k\neq j}^{|\rho|}\left(q_{t,i,k}\right)\right]\right] \\ &+ \sum_{j:\rho(j)=\rho}\left[\frac{1}{|\rho|^2}\left[\sum_{j=1}^{|\rho|}\left(q_{t,i,j}^2\right) + \frac{1}{|\rho|}\sum_{k\neq j}^{|\rho|}\left(q_{t,i,k}q_{t,i,j}\right)\right]\right]\end{aligned} \tag{1}$$

For the expectation, we have

$$\mathbb{E}\left[(|\rho|-1)\hat{w}_{t,i,\rho}\right] = \left(1 - \frac{1}{|\rho|}\right)\sum_{j:\rho(j)=\rho}\operatorname{var}\left(q_{t,i,j}\right) \tag{2}$$

Since the variance of $t_{i,j}$ is $\hat{w}_{t,i,\rho} = \dfrac{1}{(|\rho|-1)}\sum_{j:\rho(j)=\rho}\left[\dfrac{t_{i,j}}{\hat{s}_{t,j}\hat{e}_{i,\rho(j)}} - \bar{q}_{t,i,\rho}\right]^2$. Then, the variance of $q_{t,i,j}$ is

$$\operatorname{var}\left(q_{t,i,j}\right) = \frac{\mu_{t,i,j} + \left(e_{i,\rho(j)}s_{t,j}\right)^2\upsilon_{t,i,\rho(j)}}{\left(\hat{e}_{i,\rho(j)}\hat{s}_{t,j}\right)^2} = \frac{\hat{q}_i\hat{p}_{i,\rho(j)}}{\hat{e}_{i,\rho(j)}\hat{s}_{t,j}} + \upsilon_{i,\rho(j)} \tag{3}$$

We defined previously $z_{t,i,\rho} = \dfrac{\hat{q}_i\hat{p}_{i,\rho(j)}}{|\rho|}\sum_{j:\rho(j)=\rho}\left(\dfrac{1}{\hat{s}_{t,j}\hat{e}_{i,\rho(j)}}\right)$. After combining (2) and (3), we have,

$$\mathbb{E}\left[\hat{w}_{t,i,\rho} - z_{t,i,\rho} - \upsilon_{t,i,\rho}\right] = \mathbb{E}\left[\frac{\hat{q}_i\hat{p}_{i,\rho(j)}}{\hat{e}_{i,\rho(j)}\hat{s}_{t,j}} - \frac{\hat{q}_i\hat{p}_{i,\rho(j)}}{|\rho|}\sum_{j:\rho(j)=\rho}\left(\frac{1}{\hat{s}_{t,j}\hat{e}_{i,\rho(j)}}\right)\right] = 0 \tag{4}$$

So, $\left(\hat{w}_{t,i,\rho} - z_{t,i,\rho}\right)$ is an unbiased estimator for $\upsilon_{t,i,\rho}$.

Reference

1. Anders S, Huber W: Differential expression analysis for sequence count data. *Genome biol* 2010, 11(10):R106.
